# Supplementary material for: Modeling glioblastoma heterogeneity as a dynamic network of cell states
Source: Mol Syst Biol. 2021 Sep 16;17(9):e10105. doi: 10.15252/msb.202010105 (PMC8444284; doi:10.15252/msb.202010105)
Supplement: Supplementary file 6 — Source Data for Figure 5 [file MSB-17-e10105-s004.zip › Figure5A_sourcedata/GSEA_3071/hallmarks_stateVI.GseaPreranked.1623742557031/HALLMARK_PEROXISOME.html]

Details for gene set HALLMARK\_PEROXISOME[GSEA]

|  || Dataset | state63071 |
| Phenotype | NoPhenotypeAvailable |
| Upregulated in class | na\_neg |
| GeneSet | HALLMARK\_PEROXISOME |
| Enrichment Score (ES) | -0.2573713 |
| Normalized Enrichment Score (NES) | -0.89057285 |
| Nominal p-value | 0.57222223 |
| FDR q-value | 0.73347485 |
| FWER p-Value | 1.0 |
Table: GSEA Results Summary

  

Fig 1: Enrichment plot: HALLMARK\_PEROXISOME      
 Profile of the Running ES Score & Positions of GeneSet Members on the Rank Ordered List

  

| PROBE | GENE SYMBOL | GENE\_TITLE | RANK IN GENE LIST | RANK METRIC SCORE | RUNNING ES | CORE ENRICHMENT || 1 | ECI2 |  |  | 105 | 0.735 | 0.0464 | No |
| 2 | ACSL4 |  |  | 548 | 0.442 | -0.0303 | No |
| 3 | MVP |  |  | 642 | 0.413 | -0.0136 | No |
| 4 | ITGB1BP1 |  |  | 765 | 0.383 | -0.0080 | No |
| 5 | CDK7 |  |  | 979 | 0.349 | -0.0310 | No |
| 6 | SEMA3C |  |  | 1071 | 0.334 | -0.0218 | No |
| 7 | PEX6 |  |  | 1263 | 0.308 | -0.0430 | No |
| 8 | ACSL1 |  |  | 1605 | 0.268 | -0.1097 | No |
| 9 | HMGCL |  |  | 1748 | 0.256 | -0.1226 | No |
| 10 | SLC23A2 |  |  | 2036 | -0.283 | -0.1729 | No |
| 11 | HSD17B4 |  |  | 2322 | -0.337 | -0.2170 | No |
| 12 | SLC35B2 |  |  | 2469 | -0.370 | -0.2194 | Yes |
| 13 | SMARCC1 |  |  | 2522 | -0.383 | -0.1944 | Yes |
| 14 | IDH1 |  |  | 2526 | -0.384 | -0.1559 | Yes |
| 15 | SLC25A4 |  |  | 2697 | -0.425 | -0.1592 | Yes |
| 16 | MSH2 |  |  | 2786 | -0.452 | -0.1372 | Yes |
| 17 | ATXN1 |  |  | 2860 | -0.481 | -0.1079 | Yes |
| 18 | IDH2 |  |  | 2966 | -0.524 | -0.0831 | Yes |
| 19 | TOP2A |  |  | 3457 | -1.020 | -0.1137 | Yes |
| 20 | CADM1 |  |  | 3581 | -1.591 | 0.0158 | Yes |
Table: GSEA details [plain text format]

  

Fig 2: HALLMARK\_PEROXISOME: Random ES distribution      
 Gene set null distribution of ES for **HALLMARK\_PEROXISOME**
